# Supervised learning method for the prediction of subcellular localization of proteins using amino acid and amino acid pair composition

**DOI:** 10.1186/1471-2164-9-S1-S16

**Published:** 2008-03-20

**Authors:** Tanwir Habib, Chaoyang Zhang, Jack Y Yang, Mary Qu Yang, Youping Deng

**Affiliations:** 1 Department of Biological Sciences, University of Southern Mississippi, Hattiesburg, MS 39406, USA; 2 School of Computing, University of Southern Mississippi, Hattiesburg, MS 39406, USA; 3 Harvard Medical School, Harvard University, Cambridge, Massachusetts 02140, USA; 4 National Human Genome Research Institute, National Institutes of Health (NIH), U.S. Department of Health and Human Services Bethesda, MD 20852, USA

## Abstract

**Background:**

Occurrence of protein in the cell is an important step in understanding its function. It is highly desirable to predict a protein's subcellular locations automatically from its sequence. Most studied methods for prediction of subcellular localization of proteins are signal peptides, the location by sequence homology, and the correlation between the total amino acid compositions of proteins. Taking amino-acid composition and amino acid pair composition into consideration helps improving the prediction accuracy.

**Results:**

We constructed a dataset of protein sequences from SWISS-PROT database and segmented them into 12 classes based on their subcellular locations. SVM modules were trained to predict the subcellular location based on amino acid composition and amino acid pair composition. Results were calculated after 10-fold cross validation. Radial Basis Function (RBF) outperformed polynomial and linear kernel functions. Total prediction accuracy reached to 71.8% for amino acid composition and 77.0% for amino acid pair composition. In order to observe the impact of number of subcellular locations we constructed two more datasets of nine and five subcellular locations. Total accuracy was further improved to 79.9% and 85.66%.

**Conclusions:**

A new SVM based approach is presented based on amino acid and amino acid pair composition. Result shows that data simulation and taking more protein features into consideration improves the accuracy to a great extent. It was also noticed that the data set needs to be crafted to take account of the distribution of data in all the classes.

## Background

Subcellular localization is a key functional characteristic of proteins. Each protein has some elementary functions. To co-operate for such physiological function, proteins must be localized to the correct intra- or extracellular compartments in a soluble form or attached to a membrane [1]. Although the subcellular location of a protein can be determined by conducting various locational determination experiments, it is time consuming and costly to acquire the knowledge solely based on experimental measures. The number of protein sequence entry is increasing rapidly; it is highly desirable to develop a theoretical method for fast and accurately predicting protein subcellular location. A number of automated systems have been developed to predict the subcellular localization of proteins. Most of these methods for prediction of subcellular localization of proteins are based on signal peptides [2], the location by sequence homology, and the correlation between the total amino acid compositions of proteins using artificial neural network (ANN) [3]. Prediction schemes rely upon the identification of a key sorting signal (e.g. signal peptide, mitochondrial targeting signal or nuclear localization signal), the presence of which suggests a fairly unambiguous subcellular localization. von Heijne [4] and colleagues have developed subcellular localization predictors designed to identify either signal peptides or chloroplast transit peptides. According to Claros *et al*., 1997; Nakai and Kanehisa, 1992 [5], biological implication is the merit of such predictors because newly synthesized proteins are governed by an intrinsic signal sequence to their destination, whether they are to be translocated through a membrane into a particular organelle, or to become integrated into the membrane [6]. The utility of such localization prediction is dependent upon the availability of accurate N-terminal sequence, which can be somewhat problematic if the method predicts the start codons correctly, but can lead to leader sequences being missing or partially included, thereby confusing the algorithms depending on them [3]. Subcellular localization can often be assigned by searching for homologous sequences. Several localization predictors consider compartmentalizing proteins on the basis of amino acid sequence composition – correlating a typical amino acid composition with localization to a particular subcellular compartment or organelle [7]. Unfortunately, his studies of the predictive power of amino acid compositional data for subcellular localization were restricted to small sets of only a few hundred proteins. Studies showed that classifying into 12 different groups according to their subcellular locations improves the prediction accuracy. On the basis of this classification a covariant discriminant algorithm was proposed [8] to predict subcellular location of a query protein. This method is also based on amino-acid composition and the results obtained through self-consistency, jacknife and independent dataset tests indicate the improved accuracy rate.

In this paper, an attempt has been made to improve the prediction accuracy of subcellular localization of proteins using support vector machine (SVM). Two feature vectors e.g. Amino acid compositions and amino acid pairs (di-peptide) composition is considered for the prediction.

## Results

### SVM kernel selection

It is not known beforehand which kernel function, value for parameters γ, *C*, and *d* is the best for classification problems, and consequently some kind of parameter search must be done to identify the good pair of *C,* γ for RBF and *C*, *d* for polynomial and linear kernels to achieve high training accuracy. When training the SVM, we need to select the proper kernel function and the best values for the kernel parameters for the optimal results. In this study, we begin by searching the proper kernel function. Different values of the γ parameter are used to test the performance of RBF kernel ranging from the default value (i.e. 1/number of feature vectors) to 0.1. While testing the value for γ parameter, regularization parameter *C* was kept constant to 1000. Total accuracy (TA) was computed after 10-fold cross validation. Results of total accuracies for 9943 sequences with different types of kernel functions and their parameters are summarized in Tables 1, 2 &3. Further selection search was done for the value of C with 100, 500, and 1000. It was found that total accuracy was best when *C* = 100 and γ = 0.1 (see Table 1 &2). We also examined the value of exponential parameter *d* for the polynomial and linear kernel ranging from *d* = 2 to 5. We found that the total accuracy decreases with the increase in value of parameter *d* (see Table 3). Finally, to avoid the over-fitting and capturing a better decision boundary, γ = 0.1 (for RBF), *C* = 100 (for all three kernel) and *d* = 2 (for polynomial) is used. Polynomial kernel turns to linear when the value of *d* = 1. Search results confirm that the RBF kernel performs better than polynomial and linear kernels.

**Table 1:** Performance comparison of accuracies with various values of gamma (γ) parameter used in RBF kernel in SVM.

**Location**	**γ=0.002, C=1000**	**γ=0.01, C=1000**	**γ=0.03, C=1000**	**γ=0.1, C=1000**
Chloroplast	80.18	82.10	82.47	83.32
Golgi	71.69	73.58	73.86	74.16
Vacuole	65.83	65.66	66.54	69.70
Lysosome	75.43	72.70	73.73	77.22
Peroxisome	74.63	73.86	73.87	75.76

**Table 2:** Performance comparison of total accuracies with various C value (Tradeoff between training error and margin) parameter used in RBF kernel in SVM.

**Location**	**γ=0.03, C=1000**	**γ=0.03, C=500**	**γ=0.03, C=100**	**γ=0.1, C=100**
Chloroplast	82.47	82.61	82.33	84.38
Golgi	73.86	74.44	75.01	75.46
Vacuole	66.54	66.42	65.89	69.62
Lysosome	73.73	74.19	76.37	77.92
Peroxisome	73.87	74.90	77.13	77.32

**Table 3:** Shows comparison of total accuracies with different d value (degree) parameter used in Polynomial kernel in SVM

**Location**	**d=2, C=100**	**d=5, C=100**
Chloroplast	82.57	79.74
Golgi	73.00	66.42
Vacuole	66.99	60.44
Lysosome	74.21	65.55
Peroxisome	73.32	69.39

The entire search method was also done for the dataset with 1741 protein sequences. It was found that the total accuracy is worse with the RBF kernel. It appeared that the value of γ over-fits the SVM classifier but polynomial and linear kernel worked well (see Table 4).

**Table 4:** Table shows comparison of TN*f,* TP*f* and TA with balanced and unbalanced dataset using Polynomial kernel in SVM with d = 2.

**Location**	**Balanced data**			**Unbalanced data**		
	TNf (%)	TPf (%)	TA (%)	TNf (%)	TPf (%)	TA (%)
Chloroplast	82.11	86.75	82.57	63.19	79.61	64.13
Cytoplasm	69.35	76.58	70	76.62	83.4	78.41
Cytoskeleton	77.1	81.72	77.58	70.43	53.21	70.09
E.R.	72.78	77.99	73.31	66.17	58.21	66.02
Extracellular	80.91	82.01	81.02	71.98	75.02	72.33
Golgi	72.3	79.16	73	63.11	66.67	63.25
Lysosome	74	80.77	74.21	80.25	82.74	80.23
Mitochondrion	73.52	76.42	73.82	55.45	71.94	56.12
Nucleus	79.62	82.94	79.94	84.42	86.64	84.73
Peroxisome	72.96	78.6	73.32	55.11	60.33	55.16
Plasma Membrane	72.28	74.8	73.44	93.08	90.44	92.16
Vacuole	66.82	73.82	66.99	48.9	80.67	59.18

### Amino acid composition SVMs

The SVM was provided with 20 dimensional feature vector based on amino acid compositions. RBF, Polynomial and linear kernel functions are used with the most optimal value of the parameters. The best results are achieved using RBF kernel. The value of gamma factor and regulatory parameter “C” was optimized to “0.1” and “100” respectively. The results obtained after five fold cross-validation gives the overall total accuracy of 71.8%.

### Hybrid SVM

**Figure 1:**
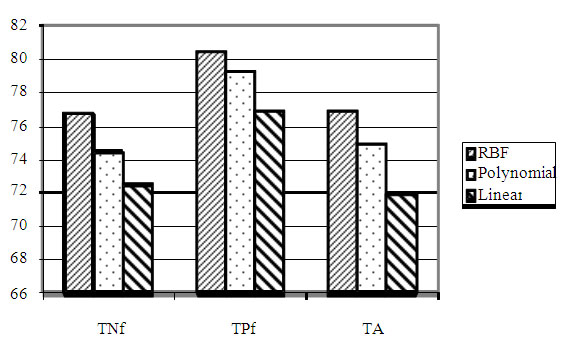
**Bar chart comparing mean true negative fraction (TNf), mean true positive fraction (TPf) and mean total accuracy (TA) for 420 features dataset with SVM using RBF, Polynomial and Linear kernel**.

**Figure 2:**
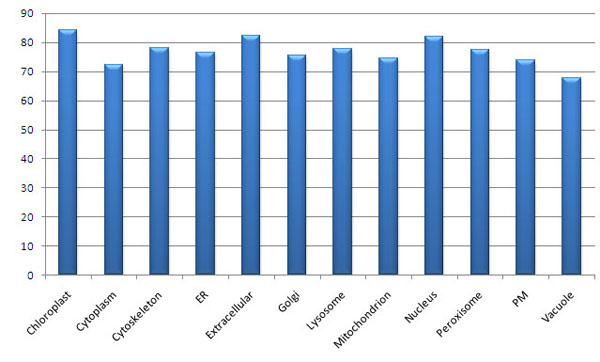
**Bar chart displaying total accuracy for 420 features dataset with SVM using RBF kernel**.

The SVM was provided with 420 dimensional (20 + (20 x 20)) feature vectors based on both the amino acid compositions and the amino acid pair compositions. In the hybrid approach best results are obtained with RBF kernel. We have also used the polynomial kernel and linear kernel but the results are poorer in comparison to RBF kernel. The value of the gamma ( γ ) and regulatory parameter C optimized to “0.1” and “100” respectively. The overall total accuracy increased to 77.0%. Figure 1 shows that RBF kernel for SVM classifier performs better than polynomial and linear kernels. Amino acid pair composition has been shown to contain sufficient information to distinguish proteins of different subcellular locations (see Figure 2).

### Feature dimensions

**Figure 3:**
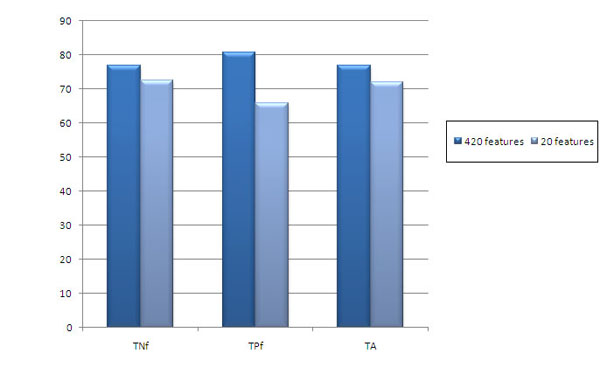
**Bar chart comparing mean true negative fraction (TNf), mean true positive fraction (TPf) and mean total accuracy (TA) for 420 and 20 features dataset with SVM using RBF kernel**.

Considering just the amino acid composition gives some information about the subcellular locations but not sufficient enough to rely on it. Adding more protein features might give better prediction accuracy. Adding physicochemical properties like polarity and charge as the additional protein feature would be able to correctly classify the proteins with average or low sequence length. To measure the role of more features for prediction, we introduced amino acid pair composition. As expected, Table 5 shows that more features give improved accuracy. Accuracy reached to 77.0% from 71.8%. It is also noticed that the variation between the overall TPf, TNf and TA is much less (see Figure 3).

**Table 5:** Prediction results of Support Vector Machine (SVM) with radial basis function (RBF) kernel. Table shows comparison of true negative fraction (TN*f)*, true positive fraction (TP*f)*, and total accuracy (TA).

**Location**	**TNf (%)**		**TPf (%)**		**TA (%)**	
	**420 features**	**20 features**	**420 features**	**20 features**	**420 features**	**20 features**
Chloroplast	83.87	66.93	88.98	85.66	84.38	68.76
Cytoplasm	71.66	77.04	78.02	43.10	72.23	73.98
Cytoskeleton	77.58	81.42	81.48	54.01	77.98	78.71
E.R.	75.98	66.26	80.74	65.50	76.45	66.18
Extracellular	82.16	78.17	83.41	68.38	82.28	77.19
Golgi	75.00	67.08	79.64	61.34	75.46	66.53
Lysosome	77.75	77.52	83.34	70.69	77.92	77.30
Mitochondrion	74.25	63.44	77.57	80.88	74.58	65.19
Nucleus	81.96	81.94	83.10	67.89	82.07	80.55
Peroxisome	77.13	67.86	80.05	59.09	77.32	67.29
Plasma Membrane	73.58	74.34	78.14	61.44	74.04	73.06
Vacuole	69.54	67.86	72.04	71.03	67.62	67.64

### Impact of number of subcellular locations

**Figure 4:**
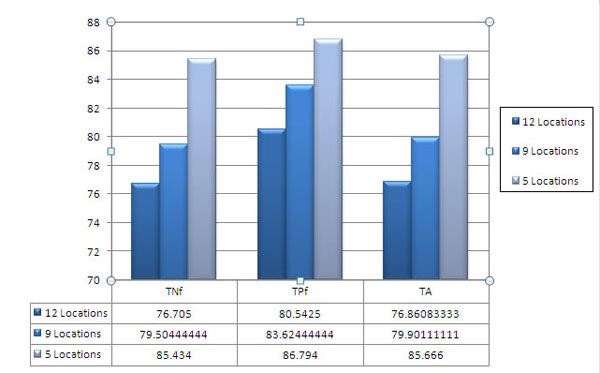
**Bar chart comparing TNf, TPf and TA for 420 features dataset using 12, 9 and 5 subcellular locations. SVM is used with Polynomial kernel**.

In order to observe the role of the number of subcellular locations on the prediction rate, two datasets were constructed. The new datasets were derived simply by removing the small subsets from the 12 locations. One dataset contains 7863 protein sequences from nine different subcellular locations. These locations are chloroplast, cytoskeleton, endoplasmic reticulum, extracellular, golgi, lysosome, mitochondrion, nucleus and peroxisome. The other dataset contains 4281 protein sequences from five different subcellular locations. Locations to which majority of proteins are targeted are considered. The locations are chloroplast, cytoskeleton, extracellular, lysosome and nucleus. Total accuracy was measured after 10-fold cross validation with RBF kernel. As it was expected, the accuracy increased significantly. Figure 4 shows that the overall total accuracy of five locations (85.7%) is higher than the overall total accuracy of nine (79.9%) and 12 locations (77.0%).

### Impact of data distribution

**Figure 5:**
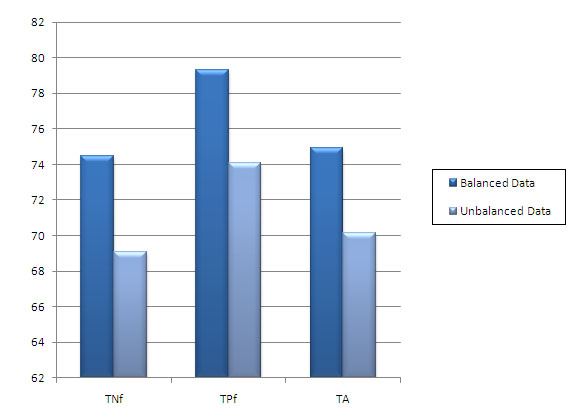
**Bar chart comparing TNf, TPf and TA for 420 features dataset with balanced and unbalanced data. SVM is used with Polynomial kernel**.

In order to check the role of the data distribution on the prediction rate, we used the dataset of 12 locations by Chou and Elrod [8]. Total of 1741 protein sequences were used in this dataset with very uneven distribution of sequences among the 12 locations (see Table 6). We found that RBF kernel overfits this dataset but polynomial and linear kernels classify well. We noted that there is large variation in TPf and TNf. On the other hand, this variation is less when the balanced dataset is used (see Table 4). Results show that few of the classes (e.g. cytoskeleton, plasma membrane) have high prediction accuracy for amino acid composition as compared to amino acid pair composition and hybrid composition. It is observed that even though the number of sequences is very low in cytoskeleton class, the average sequence length is high as compared to the other classes. Moreover there is a vast discrepancy related to sequence length in this class of sequences. Some of the sequences are very short in length while others have length few times longer than these smaller sequences. In plasma membrane class the total number of sequences is very high. These sequences are almost similar in length. This length is higher as compared to average length of most of the other classes. This results in very high total amino acid count for this class as compared to others. For both these classes, the amino acid pair composition and hybrid compositions have almost similar prediction accuracies. It can thus be inferred from these examples that the sequence length and number of sequences present in a class contribute to the amino acid composition accuracy to a greater extent. Future work is needed to assess the amino acid composition accuracy with the balanced data as compared to length and total number of sequences in each class. Overall result shows that the predictive accuracy increases as the number of sequences in each class increases. Increase in total amino acid composition for a class also leads to overall increase in accuracy for all types of compositions. The ratio of positive and negative samples in the training data might be a contributing factor for this. For the classes with less number of sequences, negative samples are very high as compared to positive samples. This results in reduced accuracies. Figure 5 shows the overall TNf, TPf, and TA with polynomial kernel. So it is important that the positive and negative samples must be balanced in training data in order to achieve high accuracy. Along with this, the total amino acid composition for all the classes used and hence all the samples also needs to be balanced for achieving good predictive accuracies.

**Table 6:** Subcellular locations and number of sequences in each location. Table shows unbalanced and balanced dataset.

**Subcellular Location**	**Unbalanced Data (No of Sequences)**	**Balanced Data (No of Sequences)**
Chloroplast	98	980
Cytoplasm	436	900
Cytoskeleton	37	991
Endoplasmic Reticulum	39	989
Extracellular	164	995
Golgi	34	997
Lysosome	37	323
Mitochondrion	76	993
Nucleus	216	992
Peroxisome	23	603
Plasma Membrane	557	996
Vacuole	24	184

### Comparison with other methods

The prediction performance of our SVM approach was compared with other methods. This comparison is summarized in the Table 7. The dataset for Neural network method [3] was based on eukaryotic and prokaryotic sequences. The sequences were divided into 3 and 4 classes and the results were obtained with 6-fold cross validation. As seen in the Table 7, the prediction accuracy of our SVM based approach for 5 classes is about 19.5% higher than Reinhardt & Hubbard's [3] approach and 6% higher than SubLoc's [13]. Our approach performs better than another method proposed by Garg *et al*., 2005 [23].

**Table 7:** Comparison of total accuracies of our method with other methods.

**Method**	**Locations**	**Accuracy (Eukaryotic / Prokaryotic proteins)**
SubLoc [13]	3	79.4/91.4
Neural Network [3]	4	66.1/80.9
Covariant discrimination	3	N.A./86.5
Markov model	4,3	73.0/89.1
HSLPRED [22]	4	77.8/N.A.
ESLPRED [21]	4	60.4/N.A.
**Our approach**	**5**	**85.6/N.A**.
Our approach	9	79.9/N.A.
Our approach	12	76.86/N.A.

## Conclusions

**Figure 6:**
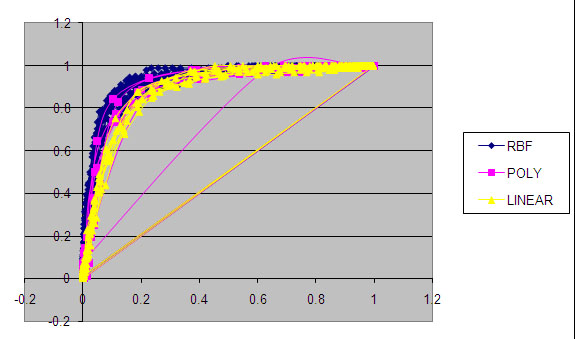
**ROC curve analysis for RBF, Polynomial and linear kernel**.

Based on the three different test results, SVM with more than one feature vector seems to be superior in accuracy for distinguish proteins of different subcellular locations. It was observed that adding more protein features improves the prediction performance. Various kernel functions with all possible values of parameters are used to train the SVM. In case of amino acid compositions, selection of value for gamma parameter ‘ γ ’, regularization parameters ‘*d’* and ‘*C’* is vital as they control the complexity of the learning process of the machine. It also influences the speed of training process. Selection of kernel function parameter is also important to define the maximum margin hyperplane. ROC curve analysis for three kernel functions indicates the area under the curve is greater for RBF kernel (0.926) as compared to polynomial kernel (0.863) and linear kernel (0.883) (see Figure 6).

## Methods

Generalization ability is important for learning algorithms because the main purpose of learning is to accurately predict unseen data. On the other hand, comprehensibility i.e. the transparency of learned knowledge and the ability to give explanation for reasoning process is also important for learning algorithm. Neural networks are the good examples of generalization ability while decision tree is with comprehensibility ability [9].

### Support Vector Machine

Support Vector Machines (SVMs), proposed by Vapnik and co-workers [10-12], are a new generation learning system based on recent advances in statistical learning theory. SVMs deliver state-of-the-art performance in real-world applications such as text categorisation, hand-written character recognition, image classification, biosequences analysis, etc. Support Vector training algorithms work with pseudocode, as well as principles of optimization, generalization and kernel theory. The construction of the support vector learning algorithm is between the “inner-product kernel” and a “support vector”. To design learning algorithm, a class of functions must be made, whose capacity can be computed. The goal of SVM is to construct a classifier that classifies the data instances in the testing data. Each instance in training data contains one class label and one feature vector. SV classifiers are based on the class of hyperplanes corresponding to decision functions. The support vectors are those data points that lie closest to the decision surface and the kernel functions are to construct optimal hyperplane, where margin of separation (i.e. closest data point) is maximized. The data points are the small subset made up of informative points. Support vector learning algorithms, which from a set of positively and negatively labelled training vectors, learn a classifier that can be used to classify the test samples. SVM learns the classifier by solving optimization problem i.e. trade-off between maximizing geometric margin and minimizing margin violations. Classifiers map the input samples into a high-dimensional feature space and seeking a hyperplane, which separates positive samples from the negative ones with the largest possible margin [13].

Linear classifier defined in feature space by

f(x)=〈w,K(x)〉+b

$$w=\sum_{i=1}^{N}y_{i}\alpha_{i}K(x_{i})$$

where sign *f*(*x*) gives prediction, *w* gives vectors which are a combination of weights α_*i*_ and labels y_i_ of the feature vectors *x_i_*.

Thus, the decision function can be written as:

$$f(\vec{x})=\mathrm{sgn}\left(\sum_{i=1}^{N}y_{i}\alpha_{i}\cdot K(\vec{x},{\vec{x}}_{i})+b\right),$$

where

$$K(\vec{x},{\vec{x}}_{i})$$

is the kernel function [14].

The popular kernel functions used in most of the SVM include the linear kernel, polynomial kernel, and the radial basis function (RBF) kernel. We need to select an appropriate kernel function and the regularization parameter C to training the SVM. The basic kernel functions are :

$$K({\vec{x}}_{i},{\vec{x}}_{j})={\left(\vec{x_{i}}\cdot {\vec{x}}_{j}+c\right)}^{d}$$

$$K(\vec{x_{i}},{\vec{x}}_{j})=\exp(-\gamma ||\vec{x_{i}}-\vec{x_{j}}|{|}^{2})$$

$$K({\vec{x}}_{i},{\vec{x}}_{j})=\tanh(\gamma x_{i}^{T}x_{j}+r)$$

Equation (i) is the polynomial kernel function with degree *d* >1, acts as a linear function when degree *d* =1. Equation (ii) is the RBF which has one parameter γ , and equation (iii) is the sigmoid function. Parameter C controls the trade-off between error and the margin, thereby bounding 0 ≤ α_*i*_ ≤ *C*.

$$C=\frac{N}{\sum_{i=1}^{N}K(x_{i},x_{j})},$$

where N is the size of the training set [13].

We have constructed twelve SVM modules to classify the proteins to particular localization. Each SVM modules is trained with all samples of one class as positive label and rest samples with negative label in 1-v-r SVMs (one-versus-rest). The goal is to construct a binary classifier or derive a decision function from the available samples. Input vector of 20 amino acid composition and 400 amino acid pair compositions is also carried out to compare the performance and the prediction accuracy.

### Amino acids and amino acid pair composition

Amino acid composition consists of only 20 components, representing the occurrence frequency of each of the 20 native amino acids in a given protein and corresponding to a 20-dimensional vector. In this study, we considered the amino acid compositions and amino acid pair composition to detect different sequence features. The feature vector ${\vec{x}}_{i}$ has 20 dimensions for amino acid compositions and 400 for amino acid pair compositions.

### Data encoding

The dataset for 12 different subcellular locations was divided into 10 subsets, each of almost equal size. The data was partitioned into training and test data. Network is trained with all samples of one class as positive label and rest samples with negative label. All modules in this method are evaluated using ten-fold cross-validation, in which the dataset was divided into ten equal size sets. The training and testing of every module is carried out ten times, each time using one specific set for testing and remaining nine sets for training. The same number of sequences for each location has been used to train the network. The prediction accuracy of SVM, BPNN and DT was examined using the confusion matrix. Total prediction accuracy (TA), true positive fraction (TPf) and true negative fraction (TNf) for each location calculated for each module to determine their performance. TA, TP fraction and TN fraction was assessed using:

$$TA=\frac{TP+TN}{TP+FP+TN+FN},$$

$$TPf=\frac{TP}{TP+FN},$$

$$TNf=\frac{TN}{TN+FP}$$

### Implementation

Two datasets with 1741 and 9943 protein sequences are used for the evaluation. The datasets were generated from version 44.3 and 44.4 of SWISS-PROT [18]. Since the neural networks are static pattern analyzers, the sequence datasets are segmented to provide much coarser representation. Sequences are grouped into 12 different cellular locations (see Table 6). Sequences with more than 40% homologs are removed using PSI-BLAST [19]. For protein sequences with the same name but from different species, only one of them was included. Protein sequences with unknown amino acid ‘X’ are not considered as we have little information about it.

### Optimization tools

Java programming language is used to generate 420 feature matrix. Adding 20 amino acids at the front and 400 dipeptides at the back, a total to 420 vectors are formed. Different methods are coded using C language to create training and testing datasets for 10-fold cross validation. SVM^light^ is used to predict the subcellular localization of proteins. The software is freely downloadable from .

All programs are implemented on Linux based PC.

## Competing interests

The authors declare that they have no competing interests.

## Authors' contributions

TH implemented the algorithm and developed the method. YD coordinated the project and revised the manuscript; YD, CZ, JY and MQY gave suggestions to improve the method and revised the manuscript. All authors read and agree to publish the manuscript.
